# Supplementary material for: The Physical Behaviour Intensity Spectrum and Body Mass Index in School-Aged Youth: A Compositional Analysis of Pooled Individual Participant Data
Source: Int J Environ Res Public Health. 2022 Jul 19;19(14):8778. doi: 10.3390/ijerph19148778 (PMC9320124; doi:10.3390/ijerph19148778)
Supplement: Supplementary file 1 [file ijerph-19-08778-s001.zip › supplementary file 1.pdf]

Supplementary file 1, Table S1. Compositional variation matrix of time spent by all participants in each intensity band.

**Intensity bands (m*g*)**	**50-100**	**100-150**	**150-200**	**200-250**	**250-300**	**300-350**	**350-700**	**≥700**
0-50	.08	.10	.13	.17	.23	.29	.43	.91
50-100		.02	.05	.10	.14	.19	.32	.78
100-150			.02	.05	.10	.15	.28	.75
150-200				.02	.06	.11	.24	.69
200-250					.02	.05	.17	.57
250-300						.02	.10	.46
300-350							.04	.37
350-700								.23

Note. Lower values reflect higher co-dependence between paired intensity bands; m*g* = milligravitational units.

Supplementary file 1, Table S2. Compositional variation matrix of time spent by boys in each intensity band.

**Intensity bands (m*g*)**	**50-100**	**100-150**	**150-200**	**200-250**	**250-300**	**300-350**	**350-700**	**≥700**
0-50	.09	.10	.13	.17	.21	.24	.33	.57
50-100		.02	.05	.08	.10	.12	.19	.40
100-150			.01	.04	.06	.09	.16	.36
150-200				.01	.03	.06	.14	.34
200-250					.01	.04	.11	.31
250-300						.01	.07	.25
300-350							.03	.21
350-700								.14

Note. Lower values reflect higher co-dependence between paired intensity bands; m*g* = milligravitational units.

Supplementary file 1, Table S3. Compositional variation matrix of time spent by girls in each intensity band.

Intensity bands (m*g*)	50-100	100-150	150-200	200-250	250-300	300-350	350-700	≥700
0-50	.08	.10	.12	.18	.24	.31	.47	1.03
50-100		.02	.05	.10	.15	.22	.37	.90
100-150			.02	.06	.11	.17	.33	.88
150-200				.02	.07	.13	.28	.81
200-250					.02	.06	.19	.67
250-300						.02	.12	.53
300-350							.05	.42
350-700								.26

Note. Lower values reflect higher co-dependence between paired intensity bands; m*g* = milligravitational units.
