# Supplementary material for: The Physical Behaviour Intensity Spectrum and Body Mass Index in School-Aged Youth: A Compositional Analysis of Pooled Individual Participant Data
Source: Int J Environ Res Public Health. 2022 Jul 19;19(14):8778. doi: 10.3390/ijerph19148778 (PMC9320124; doi:10.3390/ijerph19148778)
Supplement: Supplementary file 1 [file ijerph-19-08778-s001.zip › supplementary file 2.pdf]

Supplementary file 2, Table S4. ANOVA results of adjusted association analysis between the overall activity spectrum composition and BMI *z*-score in all participants.

	Sum of Squares	*df*	*F*	*p*
Overall activity spectrum composition	138.04	8	11.85	**< 0.001**
SES	14.10	9	1.08	0.38
Sex	15.46	1	10.62	**0.001**
Age-centred	0.04	1	0.03	0.86
Accelerometer model	2.72	2	0.94	0.30
Recording frequency	0.58	1	0.40	0.53
Residuals	2081.80	1430		

Notes. SES = socioeconomic status.

Supplementary file 2, Table S5. ANOVA results of adjusted association analysis between the overall activity spectrum composition and BMI *z*-score in boys.

	Sum of Squares	*df*	*F*	*p*
Overall activity spectrum composition	63.07	8	4.97	**<0.001**
SES	7.28	9	0.51	0.87
Age-centred	0.03	1	0.02	0.89
Accelerometer model	7.81	2	2.46	0.09
Recording frequency	4.48	1	2.82	0.09
Residuals	955.37	602		

Notes. SES = socioeconomic status.

Supplementary file 2, Table S6. ANOVA results of adjusted association analysis between the overall activity spectrum composition and BMI *z*-score in girls.

	Sum of Squares	*df*	*F*	*p*
Overall activity spectrum composition	103.15	8	9.59	**<0.001**
SES	13.83	9	1.14	0.33
Age-centred	0.05	1	0.03	0.85
Accelerometer model	0.21	1	0.15	0.69
Recording frequency	0.49	1	0.37	0.54
Residuals	1086.15	808		

Notes. SES = socioeconomic status.
