# Supplementary material for: The Physical Behaviour Intensity Spectrum and Body Mass Index in School-Aged Youth: A Compositional Analysis of Pooled Individual Participant Data
Source: Int J Environ Res Public Health. 2022 Jul 19;19(14):8778. doi: 10.3390/ijerph19148778 (PMC9320124; doi:10.3390/ijerph19148778)
Supplement: Supplementary file 1 [file ijerph-19-08778-s001.zip › supplementary file 3.pdf]

Supplementary file 3, Table S7. One-for-remaining compositional isotemporal substitutions to predict change in girls' BMI *z*-scores resulting from hypothetical time reallocations involving the 50-100 m*g* intensity band.

One-for-remaining time reallocation (min)	Predicted change in BMI z-score	Lower 95% CI	Upper 95% CI
+20	0.26	0.10	0.32
+15	0.20	0.08	0.15
+10	0.13	0.05	0.22
+5	0.07	0.03	0.11
+4	0.05	0.02	0.09
+3	0.04	0.02	0.07
+2	0.03	0.01	0.04
+1	0.01	0.01	0.02
-1	-0.01	-0.02	-0.005
-2	-0.03	-0.05	-0.01
-3	-0.04	-0.07	-0.02
-4	-0.06	-0.09	-0.02
-5	-0.07	-0.11	-0.02
-10	-0.14	-0.23	-0.06
-15	-0.22	-0.36	-0.09
-20	-0.30	-0.49	-0.12

Note. For the baseline composition, BMI *z*-score = 0.56. BMI = body mass index; CI = confidence interval.

Supplementary file 3, Table S8. One-for-remaining compositional isotemporal substitutions to predict change in girls' BMI *z*-scores resulting from hypothetical time reallocations involving the 100-150 m*g* intensity band.

One-for-remaining time reallocation (min)	Predicted change in BMI *z*-score	Lower 95% CI	Upper 95% CI
+20	-0.81	-1.34	-0.28
+15	-0.63	-1.04	-0.22
+10	-0.44	-0.72	-0.15
+5	-0.23	-0.38	-0.08
+4	-0.18	-0.30	-0.06
+3	-0.14	-0.23	-0.05
+2	-0.09	-0.15	-0.03
+1	-0.05	-0.08	-0.02
-1	0.05	0.02	0.08
-2	0.10	0.03	0.16
-3	0.15	0.05	0.24
-4	0.20	0.70	0.33
-5	0.25	0.09	0.41
-10	0.52	0.19	0.87
-15	0.83	0.29	1.38
-20	1.18	0.41	1.96

Note. For the baseline composition, BMI *z*-score = 0.56. BMI = body mass index; CI = confidence interval.

Supplementary file 3, Table S9. One-for-remaining compositional isotemporal substitutions to predict change in girls' BMI *z*-scores resulting from hypothetical time reallocations involving the ≥700 mg intensity band.

One-for-remaining time reallocation (min)	Predicted change in BMI *z*-score	Lower 95% CI	Upper 95% CI
+20	-0.97	-1.25	-0.69
+15	-0.82	-1.06	-0.59
+10	-0.64	-0.83	-0.46
+5	-0.39	-0.51	-0.28
+4	-0.33	-0.43	-0.24
+3	-0.26	-0.34	-0.19
+2	-0.19	-0.24	-0.13
+1	-0.10	-0.08	-0.15
-1	0.07	0.04	0.10
-2	0.26	0.18	0.33
-3	0.43	0.31	0.56
-4	0.68	0.48	0.87
-5	1.06	0.76	1.36

Note. For the baseline composition, BMI *z*-score = 0.56. BMI = body mass index; CI = confidence interval.

Supplementary file 3, Table S10. One-for-remaining compositional isotemporal substitutions to predict change in boys' BMI *z*-scores resulting from hypothetical time reallocations involving the ≥700 mg intensity band.

One-for-remaining time reallocation (min)	Predicted change in BMI *z*-score	Lower 95% CI	Upper 95% CI
+20	-0.77	-1.08	-0.46
+15	-0.64	-0.90	-0.38
+10	-0.48	-0.67	-0.29
+5	-0.28	-0.39	-0.17
+4	-0.23	-0.32	-0.14
+3	-0.18	-0.25	-0.11
+2	-0.12	-0.17	-0.07
+1	-0.06	-0.09	-0.04
-1	0.07	0.04	0.10
-2	0.15	0.09	0.21
-3	0.23	0.14	0.33
-4	0.33	0.20	0.46
-5	0.44	0.27	0.62
-10	1.71	1.02	2.39

Note. For the baseline composition, BMI *z*-score = 0.72. BMI = body mass index; CI = confidence interval.
